# Assessment of MRI contrast agent concentration by quantitative susceptibility mapping (QSM): application to estimation of cerebral blood volume during steady state

**DOI:** 10.1007/s10334-017-0637-9

**Published:** 2017-06-19

**Authors:** Emelie Lind, Linda Knutsson, Robin Kämpe, Freddy Ståhlberg, Ronnie Wirestam

**Affiliations:** 1 Department of Medical Radiation Physics, Lund University, Barngatan 4, 22185 Lund, Sweden; 2 Department of Radiology (Adjunct), Johns Hopkins School of Medicine, Baltimore, USA; 3 Center for Social and Affective Neuroscience, Department of Clinical and Experimental Medicine, Linköping University, Linköping, Sweden; 4 Department of Diagnostic Radiology, Lund University, Lund, Sweden; 5 Lund University Bioimaging Center, Lund University, Lund, Sweden

**Keywords:** Cerebrovascular circulation, Cerebral blood volume, Contrast agents, Magnetic resonance imaging, Magnetometry, QSM

## Abstract

**Objective:**

One major issue in dynamic susceptibility contrast MRI (DSC-MRI) is to accurately determine contrast agent (CA) concentration, since T2* relaxivity in vivo is generally unknown and varies between blood and tissue. In this study, quantitative susceptibility mapping (QSM) was used for quantification of CA concentration.

**Materials and methods:**

A DSC-MRI protocol, including phase data acquisition, was applied to 20 healthy volunteers in a test–retest study. By selecting a CSF reference region of interest (ROI), the values of all QSM images were shifted to show no CA-induced change in CSF. CA concentration and cerebral blood volume (CBV) were estimated using shifted QSM data. CSF reference ROI optimization was evaluated by investigation of CBV repeatability. The CBV age dependence was analysed and tissue T2* relaxivity was estimated.

**Results:**

The best repeatability of CBV, using an optimal CSF reference ROI, showed test-versus-retest correlations of *r* = 0.81 and *r* = 0.91 for white and grey matter, respectively. A slight CBV decrease with age was observed, and the estimated in vivo T2* relaxivity was 85 mM^−1^s^−1^.

**Conclusion:**

Provided that a carefully selected CSF reference ROI is used to shift QSM image values, susceptibility information can be used to estimate concentration of contrast agent and to calculate CBV.

**Electronic supplementary material:**

The online version of this article (doi:10.1007/s10334-017-0637-9) contains supplementary material, which is available to authorized users.

## Introduction

Provided that the relaxivities of an MRI contrast agent (CA) are known, its concentration can, in principle, be determined from the observed change in relaxation rate (Δ*R*1, Δ*R*2, or Δ*R*2*). However, the exact CA relaxivities in tissue environments are generally unknown, and relaxivites may also differ between different in vivo compartments [1]. Additionally, the relationship between the change in relaxation rate and the concentration of CA is usually assumed to be linear, but this is not always correct [2, 3]. For Δ*R*2*, measured by gradient-echo imaging, Kjølby et al. predicted that the change in transverse relaxation rate is proportional to the CA concentration in tissue, while the relationship between Δ*R*2* and CA concentration in whole blood is non-linear [1, 2]. Important applications for which the concentration of CA has to be known include quantitative perfusion and permeability measurements using CA-based techniques, i.e., dynamic contrast-enhanced MRI (DCE-MRI) and dynamic susceptibility contrast MRI (DSC-MRI).

One promising approach to avoid relaxivity-related problems is to use phase instead of magnitude information [2, 4–6]. In DCE-MRI, as well as in DSC-MRI, phase measurements in blood vessels have been used for estimations of arterial input functions and venous output functions [6–11]. The main disadvantage of using the phase of the MRI signal is that the measured phase is dependent on the geometry and orientation of the compartment of interest, and the phase shift is not generally local to the compartment containing CA [12–14]. However, for structures with simple geometries, for example, a large blood vessel (approximated by an infinite cylinder) with known angulations relative to the main magnetic field, the geometry dependence can easily be accounted for, and the underlying magnetic susceptibility of the structure can be determined. The difference in susceptibility between tissue with and without CA is proportional to the CA concentration, and the proportionality constant is described by the molar susceptibility.

In the general case, quantitative susceptibility mapping (QSM) can be used to obtain images of the magnetic susceptibility in which the geometry dependence, seen in the phase images, is removed by a deconvolution procedure [15–17]. Currently, QSM is primarily used to provide brain images with unique contrast properties, predominantly highlighting tissue with high magnetic susceptibility relative to CSF, for example, due to high iron content [18–20]. Addition of paramagnetic CAs alters the magnetic susceptibility, and QSM is thus also a promising tool for determination of CA concentration [21]. Hence, QSM has been successfully applied to estimation of CA concentration in simpler geometries, and attempts have also been made to address more complex situations in vivo [22, 23].

The aim of this study was to use QSM to quantify different levels of CA concentration in vivo. Data from a conventional DSC-MRI measurement were used to obtain phase images before, during and after a bolus passage of CA through brain tissue. Different criteria for the choice of CSF reference pixels to obtain accurate CA concentration were evaluated. By relating the difference in tissue susceptibility between pre- and post-CA conditions to the corresponding susceptibility difference (i.e., before vs. after CA passage) in whole blood, the cerebral blood volume (CBV) was assessed. The accuracy and precision of the CBV values were evaluated by comparison with literature values, age dependence and in terms of repeatability. Because CBV is a numerically well-characterized physiological parameter in normal tissue, estimation of CBV was regarded to be a useful test in the evaluation of QSM-based CA concentration quantification. The in vivo relaxivity of tissue was also estimated using susceptibility-based CA concentration estimates and the corresponding tissue ΔR2* values.

## Materials and methods

### Calculation of magnetic susceptibility using MRI phase data

For an infinite cylinder, the magnetic susceptibility can be calculated using the relationship between phase and susceptibility, according to Eq. 1, taking the angle between the cylinder and the main magnetic field into account [24]:

1 $$\chi_{\text{cyl}} = \frac{6 \phi }{{\gamma {\text{TE}}\left( {3\cos^{2} \theta - 1} \right)B_{0} }},$$

here, *ϕ* is the measured phase shift inside of the cylinder and *χ*_cyl_ is the corresponding magnetic susceptibility. Furthermore, *γ* is the gyromagnetic constant, *θ* is the angle between the cylinder and the main magnetic field and *B*_0_ is the magnetic flux density of the main magnetic field. This simple relationship between the MRI phase and susceptibility can be used to estimate the susceptibility, for example, in a blood vessel. For more complicated geometries, the magnetic susceptibility can be calculated by performing a deconvolution of the magnetic field distribution (provided by the MRI phase images) with the unit dipole field. The operation of solving this ill-posed inverse problem is often referred to as QSM, and several different approaches have been proposed to handle the deconvolution [25].

### Calculation of contrast agent concentration

If the magnetic susceptibility is known, the concentration of CA, [CA], can be determined using the molar susceptibility of the CA (in this case Gd) and the change in susceptibility induced by the CA according to Eq. 2:

2 $$\left[ {\text{CA}} \right] = \frac{{\chi_{\text{Gd}} - \chi_{0} }}{{\chi_{\text{mol}}^{\text{Gd}} }},$$

where *χ*_Gd_ is the tissue susceptibility corresponding to the calculated concentration, *χ*_0_ is the susceptibility in tissue without CA and $\chi_{\text{mol}}^{\text{Gd}}$ is the molar susceptibility of Gd.

Assuming a linear relationship between *R*2* and [CA], i.e., *R*2* = *R*2*(0) + *r*2*·[CA], an alternative measure of the concentration of CA can, in principle, be determined using the CA-induced change in R2* in combination with the transverse relaxivity *r*2*:

3 $$\left[ {\text{CA}} \right] = \frac{{R2^{ *} - R2^{*} (0)}}{{r2^{ *} }} = \frac{{\Delta R2^{ *} }}{{r2^{ *} }}, {\text{where }}\Delta R2^{*} = - \frac{1}{\text{TE}}{ \ln }\left( {\frac{S}{S(0)}} \right),$$

*S* is the magnitude signal at a given post-CA time point and *S*(0) is the baseline (pre-CA) magnitude signal. *R*2* and *R*2*(0) are the corresponding post-CA and pre-CA relaxation rates, respectively. The expression for Δ*R*2* in Eq. 3 is applicable to single-echo acquisition, assuming purely *T*2*-weighted magnitude signal. Finally, if independent estimates of [CA] and Δ*R*2* are available, *r*2* can be determined as *r*2* = Δ*R*2*/[CA] (according to Eq. 3).

### Theory of CBV estimation

CBV can be estimated using the ratio of CA concentration in a tissue compartment to CA concentration in blood. In principle, this measure provides the fraction of the tissue voxel that consists of blood, since the CA is situated only in the vascular compartment, provided that the blood–brain-barrier is intact. For plasma CAs, a correction for hematocrit (Hct) is needed in order to obtain the whole-blood volume (cf. Eq. 4). The concentration of CA is proportional to the susceptibility difference (according to Eq. 2) and the same molar susceptibility is expected in tissue and in blood, and the susceptibility difference can thus replace the concentration in the CBV calculation. Hence, CBV can be calculated using the change in tissue susceptibility between baseline level and CA steady-state conditions relative to the corresponding susceptibility change in blood, according to Eq. 4:

4 $${\text{CBV}} = 100 \cdot \frac{1}{\rho } \cdot \frac{{1 - {\text{Hct}}_{\text{LV}} }}{{1 - {\text{Hct}}_{\text{SV}} }} \cdot \frac{{\chi_{\text{tissue}}^{\text{post}} - \chi_{\text{tissue}}^{\text{pre}} }}{{\chi_{\text{blood}}^{\text{post}} - \chi_{\text{blood}}^{\text{pre}} }},$$

where *χ* represents the magnetic susceptibility (assumed to change linearly with CA concentration), *ρ* is the brain density (included to obtain CBV in units of mL/100 g) and Hct_LV_ and Hct_SV_ are the hematocrit levels in large and small vessels, respectively. Note that the magnitude of the MRI signal in voxels with 100% blood is close to the noise floor at peak concentration, due to very rapid *T*2* signal decay, implying that the MRI phase value in such a voxel is unreliable at very high concentrations. Hence, in the present study we pursued the approach of obtaining absolute values of CBV only from the CA steady-state period, instead of from the entire dynamic curve as was proposed in previous studies [22].

To illustrate how the relaxivity issue influences the CBV results, a corresponding steady-state CBV estimation based on Δ*R*2* instead of magnetic susceptibility was performed [26, 27]. Under the assumption of a linear Δ*R*2*-vs-[CA] relationship (cf. Eq. 3) and equal *r*2* levels in tissue and blood, CBV was calculated according to ${\text{CBV}} = [100\left( {1 - {\text{Hct}}_{\text{LV}} } \right)\Delta R2_{\text{tissue}}^{*} ]/[\rho \left( {1 - {\text{Hct}}_{\text{SV}} } \right)\Delta R2_{\text{blood}}^{*} ].$

### Measurements

Measurements were carried out using a 3T whole-body MRI unit (Philips Achieva, Philips Medical Systems, Best, the Netherlands). The in vivo experiments included 20 healthy volunteers, 25–84 years old, and each volunteer was scanned on two different occasions (below referred to as visit 1 and visit 2), separated by 7–20 days. All volunteers underwent a neurological physical examination before the first visit, including basic cognitive testing, to ensure that they were cognitively normal and likely to show normal cerebral perfusion. Use of prescription drugs was an exclusion criterion. Each volunteer gave written informed consent and the project was approved by the Regional Ethical Review Board in Lund, Sweden. The present study included new and entirely independent analysis of images that were originally collected in connection with a separate project [28].

Gd-based CA (0.1 mmol/kg body weight, Dotarem, Guerbet, Paris, France) was injected at a rate of 5 mL/s followed by a saline flush. Dynamic single-shot 2D GRE-EPI magnitude and phase imaging were performed with the following parameters: TE = 29 ms, flip angle = 60°, voxel size = 1.72 × 1.72 × 5 mm^3^, FOV 220 × 220 mm^2^, 20 slices and 1 mm slice gap, 70 dynamics and temporal resolution 1.24 s (covering a time period of 1 min and 26 s). The phase-encoding direction bandwidth was approximately 2600 Hz over the FOV. Before the DSC-MRI experiment, a pre-bolus of 0.02 mmol/kg body weight CA was injected as part of a separate study [28].

For one volunteer, one of the measurements was discarded due to insufficient information about the positioning of the subject relative the main magnetic field. The DICOM header information was lost during the transfer of images from the MRI scanner, and it was thus not possible to perform QSM calculations.

### Calculation of quantitative susceptibility maps

All calculations were performed using MATLAB (The MathWorks, Natick, MA, USA). Phase images were unwrapped using an algorithm that unwraps the less noisy regions first [29]. Because of the rather long TE, the unwrapping algorithm failed in some areas and, in areas with obvious remaining wraps, additional unwrapping was performed by manually adding or subtracting 2*π*. The unwrapped phase images were then filtered using a projection onto dipole field (PDF) algorithm [30, 31]. Together with the corresponding magnitude data, the unwrapped and filtered phase images were then used for calculating QSM images using the Morphology Enabled Dipole Inversion (MEDI) algorithm [21, 30, 32, 33]. As a default value, the regularization parameter *λ* was set to 300 (other *λ* settings were also evaluated, as further described below). Masks defining the boundaries of the brain, used in both the PDF algorithm and in the QSM calculations, were drawn in ITK-SNAP (http://www.itksnap.org) [34]. The segmentation tool was used in combination with manual modifications to assure inclusion of most of the brain and the sagittal sinus within the masks.

A given QSM image provided by the MEDI algorithm displays the relative distribution of susceptibility values in different tissues and regions, but the absolute susceptibility level of the QSM image is not necessarily correct, because information about the absolute phase is lost during the PDF and QSM post-processing. Furthermore, the original phase information depends not only on magnetic field variations related to the object, but also on any field change (e.g., scanner *B*_0_ drift) occurring during the dynamic scanning. To be able to compare quantitative susceptibility values among different time points of the dynamic study, the QSM image values at each time point need to be shifted to a common reference. In CA-enhanced QSM applications, all perfused tissues are dynamically affected by the CA, and a reference tissue with constant susceptibility is thus more challenging to find, compared with other QSM applications. In this study, CSF was used as a reference to establish the absolute level of susceptibility.

To objectively identify appropriate CSF reference pixels, a number of criteria were identified and applied:

- First, a region of interest (ROI) was drawn manually, including most of the lateral ventricles, defining the CSF volume of interest. The subsequently selected CSF reference pixels had to be a subset of this ROI.
- In the magnitude images, selected reference pixels were required to show a small range of signal values during the passage of CA (given by the difference between the maximum and the minimum value). The number of pixels to be selected was determined by the threshold thresh_range_, i.e., thresh_range_ decided the fraction of the manually drawn CSF ROI that was accepted as final CSF reference pixels on grounds of sufficiently low signal range. Increasing thresh_range_ implied that a larger signal range was accepted and that a larger number of CSF reference pixels were selected.
- If a single CSF pixel candidate was applied, by itself, as the CSF reference, the resulting (shifted) QSM curve of the whole volume included in the brain mask was required to show a similar tail-to-peak ratio of the dynamic curve as the result of the other CSF pixel candidates (defined as the pixels included in the manually drawn CSF ROI). The tail was defined as the five last time points in the series and the peak as the five time points including and surrounding the point where the magnitude image whole-brain ROI showed its lowest value. The threshold thresh_tailpeak_ determined the fraction of the manually drawn CSF ROI that was excluded due to either too high or too low tail-to-peak ratio values. Increasing thresh_tailpeak_ implied that a larger number of extreme (high or low) tail-to-peak ratios were discarded and that a smaller number of CSF reference pixels were selected.

The two first criteria are similar to those used in previous studies [22]. Because QSM images tend to show computational artefacts, especially in the CSF, the last criterion was added to avoid inclusion of pixels clearly affected by artefacts in the CSF reference candidate region, giving unreasonable shapes of the shifted dynamic curves.

### CBV estimation

For CBV estimation, the effect of the CA on the susceptibility in voxels containing 100% blood is required (cf. Eq. 4), and the superior sagittal sinus (SSS) was regarded to be the most reasonable candidate for obtaining pixels with 100% blood, due to minimal partial volume effects. PDF-filtered phase images and QSM images are, however, less reliable near edges of the brain [31]. Hence, susceptibility values for blood were based on phase values in manual ROIs, including three pixels in the SSS, from unwrapped (additional manual unwrapping if needed) and non-filtered phase images (i.e., not processed by the PDF filter). However, the unfiltered phase images suffer from effects of *B*_0_ drift during the scan, and the CSF pixels previously identified for QSM shifting were employed to correct for this bias. Assuming that the CA will not affect the phase in CSF, the phase data of each time point during the dynamic series was shifted so that no phase change in CSF was observed during the CA passage. The SSS phase values resulting from the shifted phase images, i.e., corrected for B_0_ drift, were converted to susceptibility values according to Eq. 1.

Additionally, susceptibility values were recorded in white matter (WM) and grey matter (GM), as well as in the previously mentioned whole-brain mask, for all time points during the dynamic series. The WM and GM ROIs were based on segmentation of magnitude images from the first time point using ​*new segment* in SPM8 (http://www.fil.ion.ucl.ac.uk/spm/software/spm8/) with a threshold level of 0.55, and susceptibility values were obtained from the shifted QSM images. Once the magnetic susceptibilities of tissue and blood were extracted, CBV was estimated according to Eq. 4, using *ρ* = 1.04 g/mL, Hct_LV_ = 0.45 and Hct_SV_ = 0.25 [35]. After visual inspection of the time curves, mean values of time points 5–14 in the dynamic series were used to establish *χ*_pre_ and mean values of the 4–10 latest time points were used for *χ*_post_, depending on when CA steady-state was reached, for both tissue and blood.

For the Δ*R*2*-based steady-state CBV estimation, Δ*R*2* was calculated according to Eq. 3 and the same WM and GM ROIs as described above were used to estimate tissue Δ*R*2*. Blood Δ*R*2* values were extracted from SSS pixels, selected according to criteria set to avoid competing T1-enhancement effects caused by the CA.

### Evaluation of CSF reference pixels for shifting the QSM image values

To establish an optimal choice of thresholds (thresh_range_, thresh_tailpeak_), used to define appropriate CSF reference pixels, whole-brain mask CBV repeatability was studied using test–retest data. The repeatability of the resulting CBV values, using a range of threshold values, was studied in terms of the results from an intra-class correlation coefficient (ICC) analysis of CBV(visit 2) versus CBV(visit 1) data (with requirement of absolute agreement in the ICC calculation), as well as in terms of the absolute levels of CBV. The default values used were thresh_range_ = thresh_tailpeak_ = 25% and the values of thresh_range_ and thresh_tailpeak_ were subsequently varied separately, one at a time, over the ranges 5–100% and 0–45%, respectively. By considering the ICC and the absolute level of CBV, the optimal choices of thresh_range_ and thresh_tailpeak_ were determined.

### Evaluation of concentration and CBV estimations

The credibility of the final susceptibility-based CBV values (assessed separately in WM and GM) obtained with the optimal threshold values for the CSF reference (i.e., thresh_range_ = 10% and thresh_tailpeak_ = 30%, see Results section), was evaluated by analyzing absolute values, repeatability and age dependence. Repeatability was assessed from a CBV(visit 2) versus CBV(visit 1) scatter plot (with linear regression and ICC analysis), as well as a Bland–Altman plot, and the age dependence was evaluated using linear regression analysis. For comparison, CBV values based on steady-state Δ*R*2* were evaluated by analyzing the absolute levels of CBV, as well as Bland–Altman plots of the test–retest data.

### Relaxivity estimations

The in vivo tissue *T*2* relaxivity (*r*2*) of the CA was determined according to *r*2* = Δ*R*2*/[CA], where Δ*R*2* was calculated according to Eq. 3 and the CA concentration was independently estimated from QSM images (*λ* = 300, thresh_range_ = 10% and thresh_tailpeak_ = 30%) using the molar susceptibility 308 ppm/M [36]. S(0) and $\chi_{0}$ were determined here as the mean signal and the mean susceptibility, respectively, of time points 5–14 in the dynamic series. The whole-brain mask defined in the QSM calculations was used as a tissue ROI. Values from time point 15 to the last time point of the dynamic series from all successful experiments were pooled together (excluding the first four time points due to saturation effects and time points 5–14 since they were used to define zero concentration), and the Δ*R*2* versus [CA] data points were analyzed by linear regression analysis and *r*2* was given by the slope.

### Evaluation of regularization parameter

Finally, the regularization parameter *λ* in the MEDI algorithm is known to affect the image appearance (e.g., image contrast), and the setting could thus affect the quantification of CA concentration in WM and GM and thereby the CBV estimates. In order to illustrate that QSM for quantification purposes may require additional considerations in the selection of λ, the difference between GM and WM CBV was investigated for different settings of *λ* (100, 300, 1000, 3000, and 5000). The CBV difference was studied instead of the ratio in order to avoid any dependence on the selected CSF reference.

## Results

**Fig. 1:**
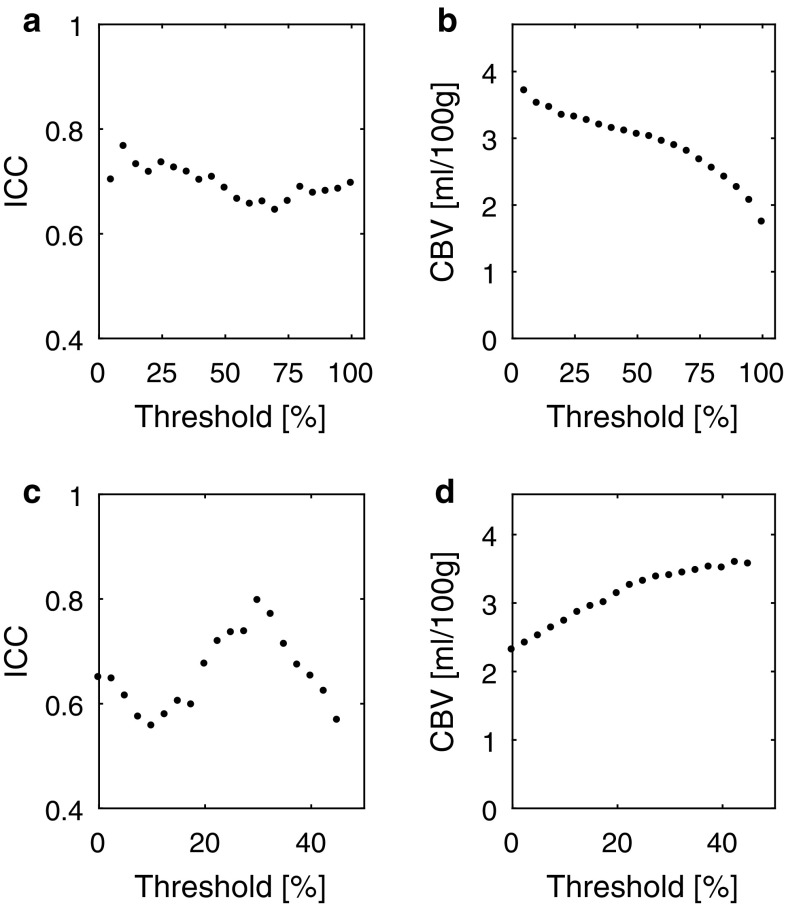
Evaluation of how (**a**, **b**) thresh_range_ and (**c**, **d**) thresh_tailpeak_ (used in the selection of CSF reference pixels) affect the repeatability and absolute levels of whole-brain CBV. The obtained ICC of test–retest CBV data is shown for **a** varying thresh_range_ and **c** varying thresh_tailpeak_. The absolute CBV (mean values from 19 volunteers) dependence of the choice of **b** thresh_range_ and **d** thresh_tailpeak_is also displayed

The results from the analysis of optimal thresholds in the CSF reference selection procedure can be seen in Fig. 1. As shown by the ICC-based optimization, a reasonably low value of thresh_range_ is beneficial, and a low value implies that only pixels showing a very small effect of the CA in the magnitude images are included in the CSF reference, and it is thus not unreasonable to select as low a threshold as possible. However, one must still ensure to include a high enough number of pixels to achieve a reasonably low variance of the mean from a statistical point of view. In subsequent analyses of CA concentration and CBV, thresh_range_ was chosen to be 10%. Regarding the tail-to-peak ratio criterion, the ICC data suggested a thresh_tailpeak_ around 25–35%. For further analysis, the value of 30% was used for thresh_tailpeak_.

**Fig. 2:**
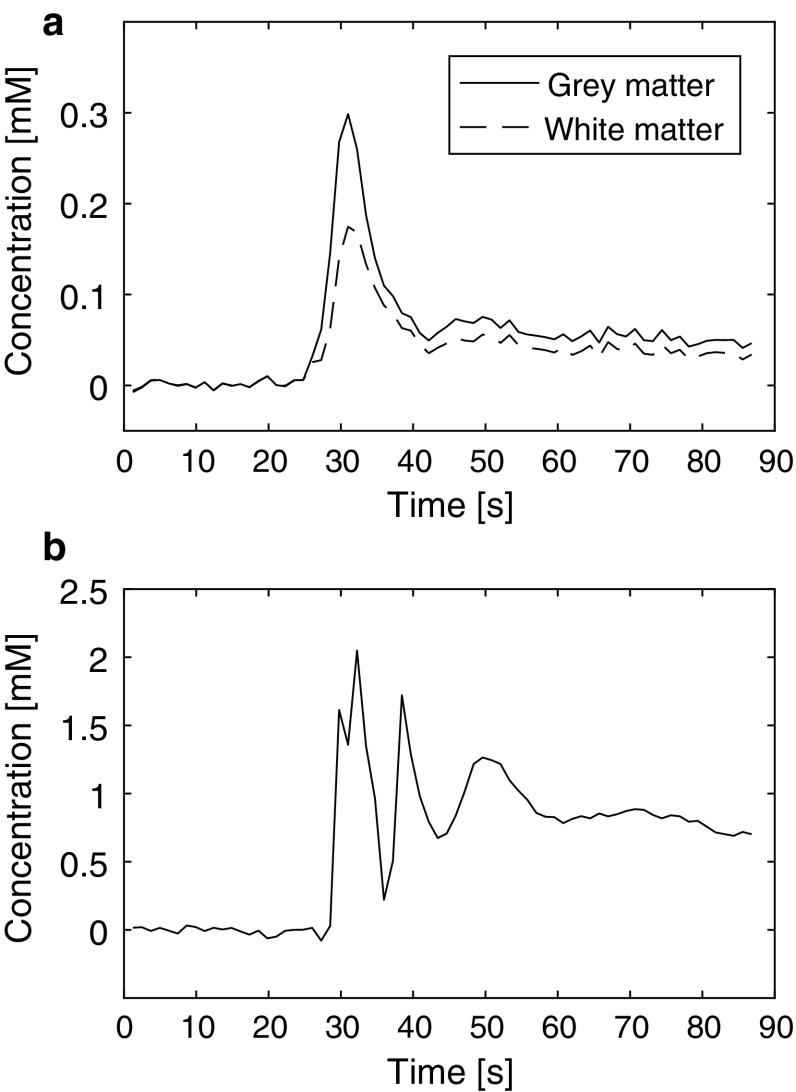
Examples of concentration curves from the dynamic contrast-enhanced measurements in **a** grey and white matter and **b** blood from one volunteer. Note that distortions at peak concentration in blood are expected

**Fig. 3:**
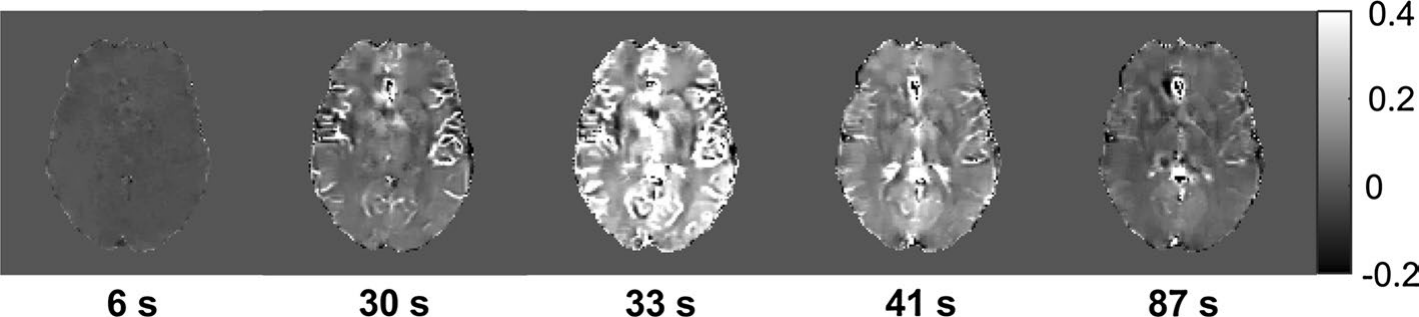
Maps showing concentration of contrast agent at different time points during the dynamic data acquisition. The same window setting is used for all time points and the values are given in mM

Representative examples of dynamic QSM data from one volunteer are shown in Fig. 2, including time curves from WM, GM and blood. Note that peak concentration values in blood are expected to be distorted due to signal saturation, pixel shift and insufficient phase unwrapping. In Fig. 3, examples of QSM-based concentration images from five different time points are displayed.

**Fig. 4:**
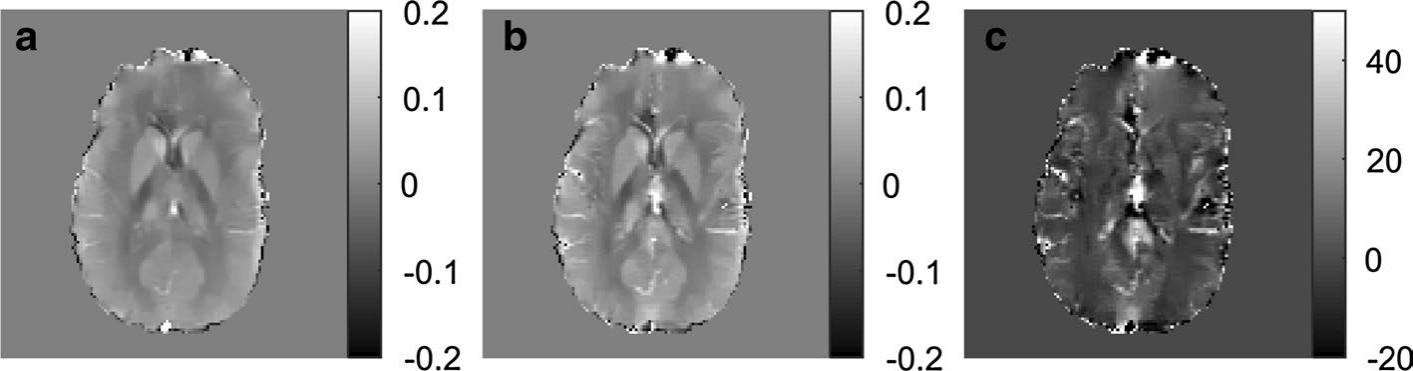
Example of QSM images **a** before CA administration, **b** after CA administration and **c** CBV map based on the two QSM images as well as information about the susceptibility difference in blood. The QSM images are shown in values of ppm and the CBV map is shown in mL/100 g. For the QSM calculations, the regularization parameter *λ* = 300 was used, and for selection of CSF reference pixels, thresh_range_ = 10% and thresh_tailpeak_ = 30% were employed

In Fig. 4, examples of QSM images before and after the CA passage, shown in ppm, are displayed, as well as a CBV image calculated according to Eq. 4. Comparing the two QSM images, the contrast between WM and GM is changed in the post-CA images, and the susceptibility in large vessels is clearly increased. In general, the CBV image shows, as expected, higher values of CBV in GM than in WM.

**Fig. 5:**
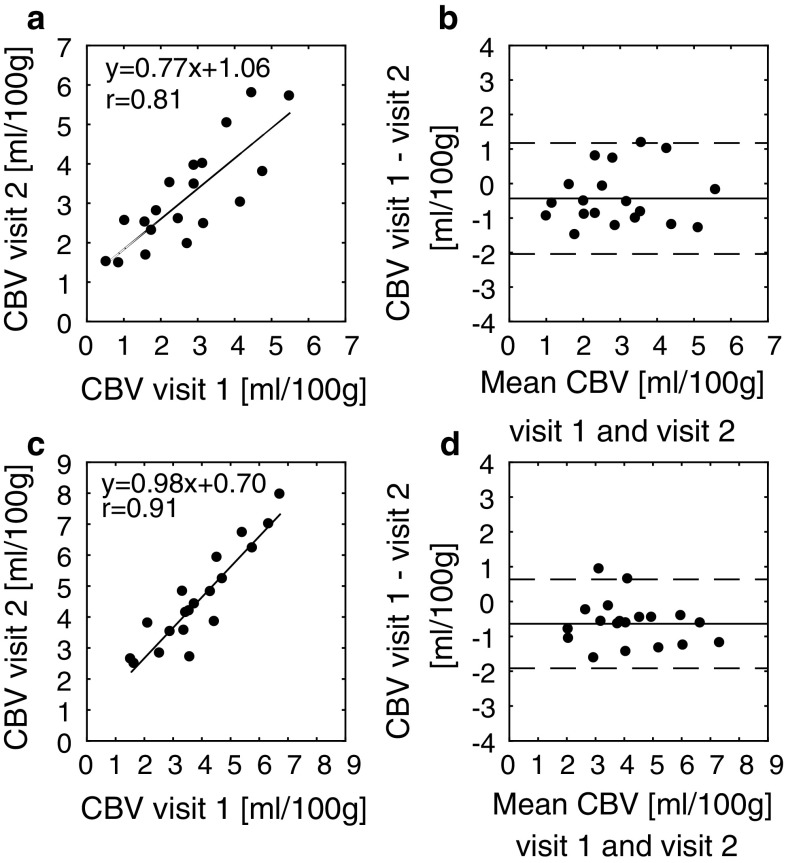
CBV values in **a**, **b** WM and **c**, **d** GM from the test–retest study with optimal thresholds. Scatter plots as well as Bland–Altman plots are shown. In the scatter plots, results from a linear regression analysis are included. The intraclass correlation coefficient analysis (under the requirement of absolute agreement) resulted in ICC = 0.78 for WM and ICC = 0.84 for GM. The *solid line* in the Bland–Altman plots represents the mean value of the difference between visit 1 and visit 2 for all volunteers, and the *dotted lines* show the mean value ± 1.96 SD of the difference between the two occasions

The results from the CBV repeatability study with optimal threshold values are shown in Fig. 5, where CBV data from WM and GM are provided separately. Scatter plots, as well as Bland–Altman plots show quite reasonable repeatability. The mean ± standard deviation (SD) of CBV from all successful measurements (*n* = 39) was 2.93 ± 1.33 mL/100 g in WM and 4.22 ± 1.52 mL/100 g in GM, and the observed GM-to-WM CBV ratio was 1.55 ± 0.34. The mean ± SD of CBV in the mask defining whole brain was 3.64 ± 1.51 mL/100 g. Obtained CA concentrations in steady-state for GM, WM and blood, as well as CA-induced changes in susceptibility are shown in Table 1, together with approximate expected values based on theoretical considerations.

**Table 1:** Experimental steady-state CA concentration and magnetic susceptibility estimates compared to expected theoretical values

	CA concentration in this study (mM) (mean ± SD)	Expected CA concentration according to theory (mM)	$\Delta\chi$ in this study (ppm) (mean ± SD)	Expected $\Delta\chi$ according to theory (ppm)
Blood	0.65 ± 0.19	0.6–1	0.20 ± 0.06	0.17–0.34
White matter	0.026 ± 0.012	0.02–0.03	0.0079 ± 0.0037	0.005–0.009
Grey matter	0.037 ± 0.012	0.03–0.06	0.011 ± 0.004	0.009–0.019

The expected theoretical values are based on an assumed concentration range of 5–10 mM at peak concentration in blood [36] and a peak-to-tail concentration ratio of 9 [41]. For WM and GM concentrations, the relation between blood and tissue is based on Eq. 4, assuming literature CBV values of 1.91 mL/100 g in WM and 3.85 mL/100 g in GM [37]. The employed CA molar susceptibility was 308 ppm/M [36]

For the ΔR2*-based CBV measurements, the mean ± SD estimates (*n* = 39) in GM and WM were 14 ± 7 mL/100 g and 7.4 ± 3.6 mL/100 g, respectively. Bland–Altman plots of the ΔR2*-based CBV test–retest results are available in Online Resource 1.

**Fig. 6:**
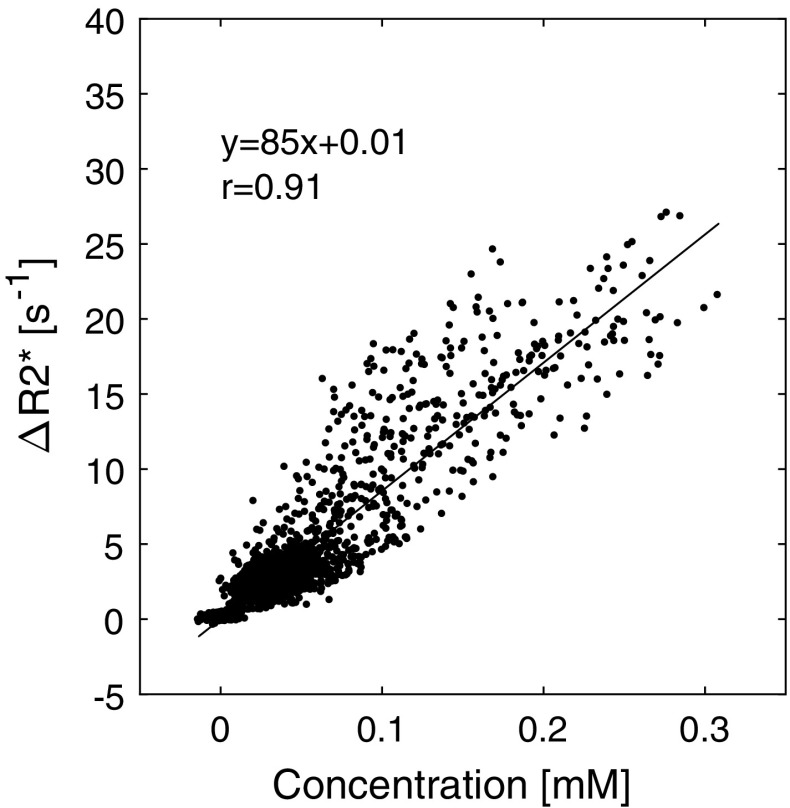
In vivo tissue CA concentration estimates from QSM images compared with the corresponding Δ*R*2* values. The results from a linear regression analysis is also shown, where the slope represents the *T*2* relaxivity of tissue, i.e. *r*2* = 85 mM^−1^s^−1^

The result from the tissue relaxivity investigation is shown in Fig. 6, giving a *r*2*_tissue_ value of 85 mM^−1^ s^−1^. The age dependence investigation indicated that CBV decreased as a function of age in both GM and WM. The linear regression analyses of CBV (*y*, in mL/100 g) versus age (*x*, in years) resulted in *y* = 4.9 − 0.025*x* (*r* = −0.37) for GM and *y* = 5.6 − 0.029*x* (*r* = −0.41) for WM.

Finally, the difference between CBV in GM and WM, for different settings of λ in the QSM calculations, is shown in Table 2. As *λ* increases, the difference between GM and WM increases, which would also lead to an increased GM-to-WM CBV ratio, provided that the required shift of the QSM images is of the same order of magnitude for all values of *λ*.

**Table 2:** Difference between CBV values in GM and WM for different regularization parameters used in the QSM algorithm

Regularization parameter in QSM algorithm, λ	CBV GM–CBV WM (mL/100 g)
100	0.9
300	1.3
1000	1.7
3000	1.9
5000	1.9

The difference in CBV indicates that calculated QSM images are smoother (i.e., contrast is lower) for lower *λ* values

## Discussion

The findings of this study suggest that reasonable estimates of CA concentration can be obtained by measuring the CA-induced change in magnetic susceptibility. Mean steady-state concentrations of CA in blood, as well as in tissue were in general agreement with expected values. The test–retest CBV analysis showed promising repeatability, and the absolute levels were in reasonable agreement with earlier studies, although the GM-to-WM CBV ratio was somewhat lower than expected [37]. Furthermore, the decrease in CBV with age supports the conclusion that these measurements actually reflect CBV [38]. Based on the fact that CBV is a well-characterized test parameter, the present results indicate that QSM is a promising approach for in vivo CA concentration estimation.

One of the major challenges in obtaining reliable, quantitative magnetic susceptibility estimates using QSM is the need of a reference region. Since almost all tissue is affected by the CA, CSF in the ventricles was chosen as a reference region in this study. Unfortunately, CSF also suffered from more artifacts than other tissue, which might be due to pulsations in the ventricles and in neighbouring vessels. To ensure the inclusion of reliable CSF pixels as a reference, a number of criteria were used in the CSF pixel selection procedure. Since the resulting CA concentration values are directly dependent on the selected CSF reference, and thus directly influence the CBV estimates, the CBV repeatability as well as absolute levels of CBV was used to evaluate optimal thresholds for the employed criteria. In this study, the resulting CBV values did, in fact, show an obvious dependence on the threshold value (Fig. 1), emphasizing the need for a robust and accurate reference selection procedure. Compared with previous studies [22], an additional criterion was introduced to improve the stability of the CSF reference pixel selection procedure, i.e., tail-to-peak ratios of the dynamic QSM curves were compared to exclude pixels generating curves that differed substantially from those obtained using other CSF pixel candidates. CBV estimates obtained with thresh_tailpeak_ set to zero can be used to evaluate the importance of this additional criterion, and it is clear that inclusion of the tail-to-peak ratio criterion resulted in increased repeatability in terms of increased ICC. However, further evaluation of procedures for reference pixel selection is warranted, for example, using other datasets. An alternative approach for background field removal is to subtract pre-CA phase images from all phase images in the time series before calculating susceptibility, similar to what has been proposed in previous studies [36]. Provided that the *B*_0_ drift of the MR scanner can be handled, the retained temporal consistency of the pre-CA phase subtraction method may simplify reference selection. This approach may also improve the phase information at the edges of the brain, although one must ensure that sufficient background phase removal is achieved over the entire image.

The concentration values in steady state obtained in this study (0.026 mM in WM, 0.037 mM in GM and 0.65 mM in blood) are consistent with theoretical values (0.02–0.03 mM in WM, 0.03–0.06 mM in GM and 0.6–1 mM in blood; see Table 1 for further comparisons and details). The dynamic curves obtained in this study also correspond well with the expected values, indicating that the employed CSF reference pixels were reasonable. Accordingly, the susceptibility values obtained were also in reasonable agreement with assumed, theoretical values.

In this study, absolute values of CBV in whole brain, GM and WM, based on CA steady-state susceptibility levels, were evaluated. The estimated CBV values (2.93 mL/100 g in WM, 4.22 mL/100 g in GM and 3.64 mL/100 g in whole brain) were in reasonable agreement, but slightly higher, than literature values, and the GM-to-WM CBV ratio of 1.55 was somewhat lower. For example, Shin et al. reported reference values to be 1.91 mL/100 g in WM and 3.85 mL/100 g in GM, based on reviewed published data from positron emission tomography studies [37]. A potential source of error that is likely to affect the GM-to-WM ratio is the choice of the regularization parameter *λ*, used in the MEDI algorithm. A lower value of *λ* gives more smoothed images, whereas a higher value of *λ* tends to return QSM images with more pronounced artifacts. To minimize the artifacts, a *λ* value of 300 was used for the evaluation of CSF reference criteria, repeatability and age dependence and, hence, the QSM images gave a somewhat smoothed impression. This study verified that lower values of *λ* results in reduced susceptibility difference between GM and WM, leading to a smaller GM-to-WM CBV ratio. Additionally, WM data are likely to show lower contrast-to-noise ratio, and it cannot be completely ruled out that the QSM algorithm, with our settings, may exhibit some degree of noise-related bias. It should be noted that a given *λ* value can result in images of different quality for different data sets, and that the optimal *λ* depends on the noise level of the input data [21]. Hence, the GM-to-WM contrast is expected to show the same trend of *λ* dependence for all data sets, but the absolute value of *λ* that generates a certain contrast pattern might differ.

As an additional argument, to increase the confidence in the obtained CBV estimates, the relationship between CBV and age was studied. A decrease in CBV with age (of approximately 5–6% per decade) could be seen in both WM and GM, with a slightly larger reduction in GM. These results are in accordance with previous studies that have shown a few percent CBV decrease per decade in GM and a smaller or non-significant decrease in WM [38–40]. The intersubject variability in CBV in this study was, however, substantial compared with the relatively slow CBV decrease with age, and the linear relationships (with low correlation coefficients) should rather be viewed upon as trendlines. The results may still, however, be regarded as a positive indication, or an additional circumstantial evidence, of the credibility of the present CBV estimation method.

The high CBV values resulting from the Δ*R*2*-based steady-state CBV estimations support the previous assumption that the transverse relaxivity *r*2* is considerably higher in tissue than in blood, and these results further emphasize that non-relaxivity-based CBV estimates are indeed warranted.

Given that both Δ*R*2* from magnitude images and CA concentration based on QSM images (calculated from phase maps) were available, the in vivo relaxivity of CA in tissue could be estimated. The good correlation between Δ*R*2* and [CA] was, in itself, an encouraging observation, and the estimated *T*2* relaxivity of 85 mM^−1^s^−1^ in this study was in very good agreement with the simulations by Kjølby et al., predicting a tissue *T*2* relaxivity of 87 mM^−1^s^−1^ at 3T [1]. However, the present tissue *T*2* relaxivity estimate was higher than in a previous experimental study of in vivo relaxivity, based on a comparison with cerebral blood flow (CBF) values from a reference arterial spin labelling (ASL) method, where the *T*2* relaxivity was estimated to be 32 mM^−1^s^−1^ [41].

In this study, EPI images with limited spatial resolution, slice gap and EPI-related distortions were used for calculation of the QSM images. Despite the suboptimal conditions for susceptibility calculations, images seemed generally robust with regard to the subjective impression of image quality, as well as the quantitative values extracted from the images. However, the QSM image quality varied between different volunteers and QSM generally suffers from some degree of artifacts. In this study, data from a conventional DSC-MRI sequence was used, optimized for CA concentration estimation based on Δ*R*2* and not on QSM. In future studies, imaging parameters can be further optimized to improve conditions for QSM calculations based on phase data, for example, by using a 3D sequence to avoid slice gaps, using multi-echo acquisition to improve frequency estimations and simplify phase unwrapping. If possible, one should improve spatial resolution and increase the bandwidth (to reduce geometric distortions), for example, by using segmented EPI readout.

Except for the susceptibility difference in tissue, extracted from the QSM image, the other main component of CBV estimation was to establish the pre-CA versus post-CA susceptibility difference in blood. Since the spatial resolution in EPI is rather low, the number of vessels that can be used is limited. In this study, the SSS was employed, mainly because it is a large vessel of modest curvature with convenient location. Furthermore, veins tend to exhibit less blood pulsations than arteries, which might be of relevance when phase data are used. However, one drawback of the SSS is that it is situated close to the edge of the mask used in the calculations, which leads to uncertainties in filtered phase images and in calculated QSM images. For this reason, non-filtered phase images were used instead of QSM images to estimate the susceptibility difference in blood. When phase images are used instead of QSM images, susceptibility needs to be calculated by taking the geometry of the vessel into account, as described in the methods section. However, any curvature of a vessel may complicate the calculations, and this may have contributed to the relatively large range of CBV values seen amongst different volunteers. Furthermore, the selected blood pixels were tracked throughout the time series, i.e., any CA-induced apparent shift of the vessel position in the images, caused by the low EPI bandwidth in the phase direction, was disregarded. However, visual inspection of the phase images in which the vessel ROIs were defined ensured that the selected ROI was located within the blood compartment both before and after the CA passage. Finally, another issue concerning the susceptibility values in blood is the absolute phase level in non-filtered images. To correct the phase maps for *B*_0_ drift, the same CSF reference pixels as for QSM images were used, under the assumption that the CSF is not affected by the CA. However, even if such an assumption is true for susceptibility, the *phase* in the CSF pixels can still be affected by CA in compartments adjacent to the ventricles. Compared to the large effect of CA in whole blood this effect is, however, expected to be minor. A promising alternative to the use of phase images for estimating SSS susceptibility would be to perform QSM using the recently proposed total field inversion (TFI) approach [42], designed to improve estimation of susceptibility close to the edge of the brain.

It is generally acknowledged that DSC-MRI provides accurate relative CBV and CBF maps. However, absolute CBV and CBF values provided by DSC-MRI tend to be overestimated [43], and it would thus be of great interest to independently determine absolute values of CBV, for calibration purposes. It is sufficient for such a calibration measurement to be global or ROI-based, because DSC-MRI is assumed to show correct relative distributions. Separate CBV or CBF measurements techniques have previously been used for such calibration of DSC-MRI-based CBV and CBF levels, including Bookend, vascular space occupancy (VASO) and ASL [38, 44, 45], and analyses of repeatability and accuracy of these techniques have shown both promising and challenging outcomes. With the technique proposed in this study, data inherent to the DSC-MRI scan can be used for a separate steady-state CBV measurement without the need for any additional sequence in the protocol. In all CA steady-state CBV techniques (e.g., the present QSM approach, Bookend [37], VASO [46]), one must, obviously, ensure that the major temporal fluctuations in CA concentration (i.e., first passage and recirculation) have subsided. Thereafter, a slow clearance of CA follows, but the employed data points are acquired during only a few tens of seconds, so we have assumed that the concentration remains fairly stable during that time. Furthermore, any minor decay in tissue CA concentration during measurement, after the recirculation phase, should be more or less reflected by a corresponding decrease in the blood concentration, which would reduce the resulting error in the CBV estimate (cf. Eq. 4). Data acquisition as soon as possible after the recirculation phase is beneficial in terms of higher contrast-to-noise ratio.

If absolute CBV is sufficient, i.e., no CBF or mean transit time (MTT) is needed, an alternative would be to use high-resolution gradient-echo images before and after the CA administration to provide a measure of CBV. However, in order for this to be possible, a different approach of finding an appropriate CSF reference would be needed, since the behavior during the CA passage was used in this study to select appropriate CSF reference pixels.

Provided that phase data from a contrast-enhanced dynamic first-pass bolus tracking experiment can be used to calculate QSM images and to estimate reliable CA concentrations, dynamic QSM can also be used to calculate CBV, CBF, and MTT using the same approach as for DSC-MRI, but using QSM images for CA concentration estimations instead of magnitude data. This has been proposed in previous studies [22, 23], and is indeed a promising concept, because common problems recognized in DSC-MRI as, for example, different *T*2* relaxivities in different compartments and nonlinear CA response in whole blood would be avoided. However, in order for dynamic QSM perfusion imaging to be feasible, it is crucial that the CA concentration estimates in both artery and tissue are spatially and temporally reliable, and the present study is a first step towards such an evaluation.

## Conclusion

CBV estimations based on the susceptibility differences in tissue and blood, before and after CA administration, resulted in realistic absolute levels of CBV, as well as quite reasonable repeatability and an expected age dependence trend. The results indicated that phase and QSM imaging constitutes a promising approach for estimating tissue CA concentrations in vivo, not associated with the problems seen when using relaxivity-based CA estimations.

## Electronic supplementary material

Below is the link to the electronic supplementary material.

Online Resource 1 Bland–Altman plots of test-retest results from the ΔR2*-based steady-state CBV estimation (see details in Materials and Methods section). Solid lines indicate the mean of the difference between visit 1 and visit 2 and the dotted lines represent the mean value ± 1.96 SD. Results from (a) white matter and (b) grey matter are shown. (EPS 27 kb)
